# Temozolomide Therapy for Aggressive Pituitary Tumors: Results in a Small Series of Patients from Argentina

**DOI:** 10.1155/2015/587893

**Published:** 2015-05-27

**Authors:** Oscar D. Bruno, Lea Juárez-Allen, Silvia B. Christiansen, Marcos Manavela, Karina Danilowicz, Carlos Vigovich, Reynaldo M. Gómez

**Affiliations:** ^1^Foundation of Endocrinology, 1425 Buenos Aires, Argentina; ^2^Pathology Laboratory, Hospital Italiano, 1199 Buenos Aires, Argentina; ^3^Division of Endocrinology, Hospital de Clínicas, University of Buenos Aires, 1120 Buenos Aires, Argentina

## Abstract

We evaluated results of temozolomide (TMZ) therapy in six patients, aged 34–78 years, presenting aggressive pituitary tumors. In all the patients tested O^6^-methylguanine-DNA methyltransferase (MGMT) immunoexpression in surgical specimens was absent. Patients received temozolomide 140–320 mg/day for 5 days monthly for at least 3 months. In two patients minimum time for evaluation could not be reached because of death in a 76-year-old man with a malignant prolactinoma and of severe neutro-thrombopenia in a 47-year-old woman with nonfunctioning pituitary adenoma. In two patients (a 34-year-old acromegalic woman and a 39-year-old woman with Nelson's syndrome) no response was observed after 4 and 6 months, respectively, and the treatment was stopped. Conversely, two 52- and 42-year-old women with Cushing's disease had long-term total clinical and radiological remissions which persisted after stopping temozolomide. We conclude that TMZ therapy may be of variable efficacy depending on—until now—incompletely understood factors. Cooperative work on a greater number of cases of aggressive pituitary tumors should be crucial to establish the indications, doses, and duration of temozolomide administration.

## 1. Introduction

Aggressive pituitary tumors are invasive macroadenomas refractory to surgical and medical treatments, showing tendency to continuous growth and implicating a bad vital prognosis [1]. Until some years ago, no therapies were efficacious in treating that kind of tumors. First publications of treatment with the alkylating agent temozolomide (TMZ) appeared in 2006 [2, 3] and since then, variable responses to this drug have been reported in a limited number of cases with tumor volume reduction and control of the disease in some of them. We present here our experience with the use of temozolomide in six patients with different variants of aggressive pituitary tumors.

## 2. Patients and Methods

Six patients with intention-to-treat with TMZ, presenting different types of aggressive pituitary tumors, were evaluated. They were 5 women aged 34–52 and one 78-year-old man. They all presented macroadenomas (more than 10 mm) with cavernous sinus invasion, two of them with third par palsies and one with bitemporal hemianopsia. The only male patient had pituitary carcinoma (malignant prolactinoma) with an isolated parietal metastasis which was first biopsied and then surgically excised. All patients had had unsuccessful previous pituitary surgery (from 1 to 5 times), radiotherapy in 3, and conventional drug treatment in 4 of them, aimed at controlling hyperfunction and/or tumor volume (Table 1). The definition of aggressive pituitary tumor was based on clinical grounds (invasive macroadenomas refractory to surgical and medical treatments, showing tendency to continuous growth) as previously stated. We use the denomination* pituitary carcinoma* when extrapituitary presence of tumor (metastasis) is found. Temozolomide was administered as oral pills in variable doses, from 140 to 320 mg/day for 5 days every month, for at least 3 months before evaluating results. TMZ administration was preceded by the oral intake of ondansetron, as antiemetic prevention. Hematologic and liver function tests were performed before each cycle of therapy. Results of treatment were evaluated by monthly clinical examination and pituitary MRI after at least 3 months of therapy; computerized visual field examination and routine hormone tests were also made, when indicated.

For determinations of MGMT and marker of cell proliferation Ki67 on pathological specimens, all blocks were formalin buffer fixed and paraffin embedded. Cuts of 3-4 microns were made and stained with hematoxylin and eosin. Immunohistochemical determinations for adenohypophyseal hormones GH, FSH, LH, and TSH were made by using rabbit polyclonal Cell Marque (http://www.cellmarque.com/) antibodies whereas for PRL and ACTH, rabbit polyclonal DAKO (http://www.dako.com/) antibodies were employed. Ki67 and O^6^-methylguanine-DNA methyltransferase Ab-1 (MGMT) were measured by using mouse monoclonal antibodies from Thermo Scientific (http://www.thermoscientific.com/) in a 1 : 20 dilution. Immunostaining for MGMT was considered negative when lower than 10%.

## 3. Results

**Figure 1:**
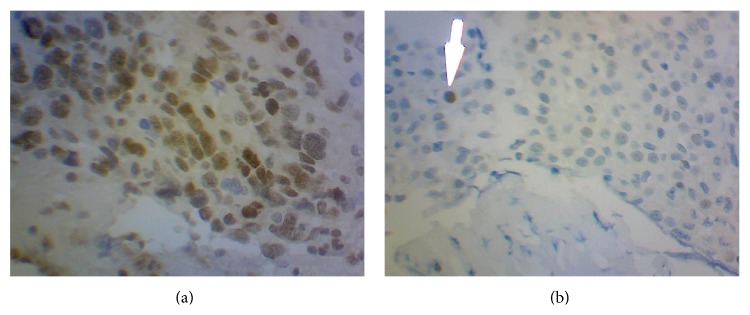
The upper panel (a) shows a diffuse positive MGMT control (glioblastoma). The lower panel (b) corresponds to a negative MGMT immunostaining of macrocorticotropinoma in patient GM.

Figure 1 shows a MGMT-negative macrocorticotropinoma study of patient GM as compared to a MGMT-positive glioblastoma.  Table 2 shows the results of MGMT and Ki67 immunohistochemistry, individual doses administered, length of therapy, and clinical outcome in the six patients.

Drug therapy effect could not be evaluated in patients JB and SA because they failed to complete a 3-month treatment. JB had a malignant prolactinoma with brain metastases which deceased after the first administration of TMZ and SA developed severe thrombocytopenia and neutropenia after the first cycle of therapy. In two more patients TMZ was stopped after 4 (LC) and 6 (DDO) months of treatment, because it was considered ineffective in reducing tumor size. The two last patients having macrocorticotropinoma and Cushing's disease have been reported in detail elsewhere [4]. They showed clinical response after just 3-4 cycles of administration of TMZ with remission of ocular signs, normalization of cortisol alterations, and significant shrinkage (more than 50%) of the tumors, which completely disappeared one year later and, most interestingly, long time (19–30 months) after stopping therapy the patients remained well with no signs of tumor relapse [4].

## 4. Discussion

Frequency of pituitary tumors appears to be higher than previously suspected, as high as 1 in 1000 of the general population [5, 6]. They are usually benign and in most cases controlled by surgery, radiation, or medical treatments. In 2004 the World Health Organization defined as “*atypical*” those tumors exhibiting a MIB-1 (Ki-67) proliferative index >3%, strong p53 immunoreactivity, and increased mitotic activity [7]. They make up 15% of resected pituitary tumors [8]. Up to 45% of macroadenomas show signs of invasion of the sphenoid or cavernous sinus [9]. The concept of  “*aggressive*” pituitary tumors represents a clinical appreciation to designate tumors that may recur quickly after surgery, grow into the cavernous sinus or skull base, and show resistance to the usual therapeutic means. The name pituitary* carcinoma* is reserved for those tumors with neural or extraneural metastases which make up less than 1% of the totality of pituitary tumors. It has to be emphasized that they do not show histological differences with other aggressive tumors save for the existence of metastases [10].

So called silent pituitary adenomas are tumors, mainly gonadotrope, corticotrope, and somatotrope, having an aggressive behavior, with frequent recurrences which made up 9% in 100 samples studied retrospectively [11]. They can be classified as “*silent*,” with immunohistochemical evidence but* no* biochemical or clinical evidence of hormone excess, or “*clinically silent*” with immunohistochemical* and* biochemical evidence but* no* clinical evidence of hormone excess.

Temozolomide is an alkylating drug which has been used mainly in the treatment of glioblastoma multiforme but also for colorectal cancer and melanoma [12–14]. This drug has been used for the treatment of pituitary carcinoma and aggressive adenoma from the year 2006 onwards [2, 3]. Its mechanism of action is through sticking an alkyl group to DNA bases, principally guanine, which induces methylation. Subsequently, it provokes the fragmentation of DNA by repairing enzymes in its attempt to replace the alkylated bases [15]. Up to now, around 105 pituitary tumors treated with TMZ have been reported in the literature with variable results (Table 3) [4, 16–53]. More than half (~60%) were aggressive adenomas, the remaining being pituitary carcinomas. Most were functioning tumors, especially corticotropinomas and prolactinomas (~80%). Global efficacy of TMZ therapy oscillated between 55% for aggressive adenomas and 58% for pituitary carcinomas, but it has to be underlined that criteria for efficacy were quite diverse, going from variable reduction to “stabilization” in tumoral size. It has to be remarked that in none of the reported cases a sustained disappearance of tumor after stopping TMZ was described. As far as aggressive macrocorticotropinomas are concerned, we were able to find 37 published cases silent or with overt hypercortisolism. Once again, criteria employed to evaluate response were quite diverse. In just one of those cases [20], the tumor disappeared under treatment but if the patient was treated with a CAPTEM schema (capecitabine plus temozolomide) we cannot know which one of the two drugs was more effective.

Doses of temozolomide usually recommended in neurology are adapted to body surface and oscillate from 150 to 200 mg/m^2^ [54]. Doses employed in our patients were variable, but generally lower than recommended. It has to be underlined that dose amount was mostly determined by availability following individual medical coverage. Interestingly, patients MC and GM who had total remission received fixed doses of 250 mg/d and 180 mg/d, respectively, while, if adapted to body surface area, those figures should have been 291–388 mg/d for MC and 273–364 mg/d for GM. The role that the DNA repairing systems may play in the effectiveness of temozolomide is controversial, especially concerning MGMT. This enzyme can reverse methylation of the guanine residues, thus antagonizing the effect of the drug. It has been reported that a low expression or the absence of this enzyme strongly correlates with the response to TMZ [15]. This has been challenged by other authors, who failed to find such a correlation [21, 23]. It has been proposed that the preservation of another enzyme system, MSH6 (DNA mismatch repair protein), correlated better with the response to TMZ than the absence of MGMT [23, 54].

In our series, the five patients in whom we were able to measure MGMT failed to show a significant expression (less than 5%); two of them having aggressive corticotropinoma had excellent clinical responses to temozolomide. Nevertheless, this does not enable us to extrapolate any conclusions at this respect, since two other MGMT-negative patients who completed the minimum period of treatment failed to show a response.

For a more rational use of TMZ several points deserve clarification: What should be the starting and maintenance doses? How can efficacy be defined? How long should the treatment be given? How big is the mutagenic risk? What is the recurrence risk after stopping TMZ? What is the probability of relapse with resistance to TMZ after stopping a successful therapy?

In conclusion, although less common, clinically aggressive pituitary tumors are not at all exceptional and pose special therapeutic challenges because surgery and radiotherapy are frequently useless and usual drug therapy is of variable and unpredictable efficacy. So called “silent” tumors appear to be particularly aggressive and, although less frequent, invasive corticotropinomas may present a difficult challenge as well, since besides local complications, they put life at risk because of the metabolic consequences of excess cortisol secretion. Temozolomide may be a salvage drug in selected cases, mainly in prolactinoma and corticotrope tumors. Cooperative work on a greater number of cases of aggressive pituitary tumors should be of the outmost importance to establish the indications, doses, and duration of temozolomide administration.

## Figures and Tables

**Table 1:** Main clinical traits of 6 patients with intention-to-treat with temozolomide.

Patient	Sex	Age	Tumor type	Number of previous surgeries	RxT	Previous drug therapy
JB	M	78	PRL Ca	1	Yes	CAB
SA	F	47	CNFPA	3	No	CAB
LC	F	34	GH-oma	2	No	CAB, SSAs
DDO	F	39	NS	2	Yes	None
CM	F	42	CD	1	No	None
GM	F	52	CD	5	Yes	KNZ

PRLCa: prolactin carcinoma; GH-oma: somatotropinoma; CNFPA: clinically nonfunctioning pituitary adenoma; NS: Nelson' syndrome; CD: Cushing's disease; RxT: radiotherapy; CAB: cabergoline; SSAs: somatostatin analogs; KNZ: ketoconazole.

**Table 2:** Results of MGMT and Ki67 immunohistochemistry, doses, length of therapy, and clinical outcome in 6 patients with intention-to-treat with TMZ.

Patient	Tumor type	MGMT	Ki67	TMZ mg/d	Months	Outcome
JB	PRL Ca	(−)	10%	140	1	Death
SA	CNFPA	ND	2%	150	1	Failure
LC	GH-oma	(−)	3%	320	4	Failure
DDO	Nelson's	(−)	1%	240	6	Failure
CM	CD	(−)	6%	250	13	Remission
GM	CD	(−)	4%	180	29	Remission

**Table 3:** Literature update on aggressive pituitary adenomas and carcinomas.

Author, year [reference]	Sex/age	Tumor type	Ki67 (%)	MGMT	TMZ (mg/m^2^) & schedule (no. cycles)	MRI (% shrinkage)	Clinical outcome
Thearle et al., 2011 [16]	M/50	ACTH SA Ad → Ca → NS	31	NA	200 × 5/28 + CAP (4)	Reduced (75)	Death

Dillard et al., 2011 [17]	M/56	ACTH Ad	5-6	NA	150–200 × 5/28 (4)	Reduced (60)	CR

Annamalai et al., 2012 [18]	M/65	ACTH Ca	5–15	Low	200 × 5/28 (15)	PR of METS	“Remained well”

Moshkin et al., 2011 [19]	M/46	ACTH SA Ad → Ca	1–5	(+)	200 × 5/28 (16)	No change	Progression

Zacharia et al., 2014 [20]	M/50	ACTH Ad	<5	(−)	150, 5/28 + CAP (30)	SD	PR
	F/46	ACTH Ad	15–20	(−)	150, 5/28 + CAP (32)	CR	CR
	M/44	ACTH Ad	<5	(−)	150, 5/28 (45) + CAP + A-SST	CR	CR

Raverot et al., 2010 [21]	M/31	ACTH Ca → NS	20	50 (+)	150–200 × 5/28	No change	NA
	M/49	ACTH Ad	20	<1	150–200 × 5/28	No change	NA
	M/38	ACTH Ca	10	30 (+)	150–200 × 5/28	SR	“Significant response”
	F/42	ACTH Ad	0.5	0	150–200 × 5/28	SR	“Significant response”
	M/32	PRL Ca	NA	NA	150–200 × 5/28 (24)	Reduced (60), disappearance of METS	NA
	M/52	PRL Ad	0.5	30	150–200 × 5/28 (8)	No change	NA
	M/54	PRL Ca	1	0	150–200 × 5/28 (5)	No change	NA
	F/30	PRL Ca	10	100	150–200 × 5/28 (3)	No change	NA

Bush et al., 2010 [22]	NA	Null cell Ad	<3	—	75 × 21/7 (10)	Reduced (20)	Stable
	NA	ACTH Ad	18	<10	75 × 21/28 (11)	Reduced (80)	“Improved”
	NA	NF Ad	<3	10–50	75 × 21/(13)	SD	SD
	NA	Null cell Ad	6	>50	75 × 21/7 (10)	SD	SD
	NA	PRL Ad	NA	<10	75 × 21/7 (11)	Reduced (80)	“Improved”
	NA	Null cell Ca	>20	>50	75 × 21/7 (2)	SD × 2 months	NA
	NA	Null cell Ca	>20	<10	75 × 21/7 (7)	Progression	Death

Hirohata et al., 2013 [23]	M/59	NF Ca	74.6	(+)	150–200 × 5/28 (5)	PR	NA
	F/42	ACTH Ca	3.4	(−)	150–200 × 5/28 (7)	PR	NA
	F/60	PRL Ca	18.7	(−)	150–200 × 5/28 (13)	CR	NA
	M/23	NF Ca	2.5	(+)	150–200 × 5/28 (7)	SD	NA
	F/53	ACTH Ca (Crooke cell)	2.0	(+)	150–200 × 5/28 (20)	CR	NA
	F/60	PRL Ca	27.8	(+)	150–200 × 5/28 (12)	PR	NA
	M/57	ACTH Ca	10	(+)	150–200 × 5/28 (8)	SD	NA
	F/73	NF Ca	5.6	(−)	150–200 × 5/28 (22)	PR	NA
	M/60	PRL Ca	40.2	(−)	150–200 × 5/28 (24)	PR	NA
	F/61	NF Ca	12.2	(+)	75 × 6 weeks + RT	Progression	NA
	F/66	PRL Ad	9.4	(−)	75 × 6 weeks + RT	CR	NA
	F/49	PRL Ad	3.9	(−)	NA (3)	Progression	NA
	F/45	ACTH Ad (Crooke cell)	46.8	(+)	150–200 × 5/28 (11)	PR	NA

Losa et al., 2010 [24]	M/64	ACTH Ad	NA	NA	150–200 × 5/28	Progression	Death
	M/52	ACTH Cd	1	(−)	150–200 × 5/28	“Response”	Required GC therapy
	F/55	ACTH Ad → NS	5	(−)	150–200 × 5/28	SD	NA
	F/53	ACTH Ad	2.5	(+)	150–200 × 5/28	Progression	No change
	M/62	PRL Ad	9	(−)	150–200 × 5/28	SD	NA
	F/57	PRL Ad	NA	Noninformative	150–200 × 5/28	“Response”	“Improved”

Moyes et al., 2009 [25]	F/64	ACTH Ad → NS	“High”	(−)	200 × 5/28 (6)	“Marked shrinkage”	“Improved”

Takeshita et al., 2009 [26]	F/46	ACTH Ca → NS	~3	<5 (−)	150–200 × 5/28 (23)	CR tumor + METS	Required GC therapy

Curtò et al., 2010 [27]	M/42	ACTH Ca	2–18	<5 (−)	150–200 × 5/28 (17)	Reduced (>90)	“Improved”

Mohammed et al., 2009 [28]	F/43	ACTH Ad	NA	(−)	150–200 × 5/28 (12)	PR	“Improved”
	M/60	ACTH Ca → NS	NA	(+)	150–200 × 5/28 (12)	PR	“Improved”

Bode et al., 2010 [29]	NA	ACTH Ca → NS	NA	NA	150 × 5/28	PR	NA

Jouanneau et al., 2012 [30]	NA	SA → Ca	NA	NA	200 × 5/28	NR	NA

Asimakopoulou et al., 2014 [31]	F/55	ACTH Ad (Crooke cell)	1	NA	150–200 × 5/28	CR	CR

Bengtsson et al., 2015 [32]	F/71	ACTH Ad	50	90	150–200 × 5/28	SD	NA
	F/31	GH Ad	7	9–100	150–200 × 5/28 (6)	Reduced (50)	Regrowth after TMZ stop
	F/13	GH Ad	5	95	150–200 × 5/28	NA	NA
	M/33	PRL-GH Ad	23	10	150–200 × 5/28 (3)	Reduced (35)	SD 25 months after TMZ
	M/22	PRL Ad	8	90	150–200 × 5/28 (15)	Reduced (25)	Death
	M/34	PRL Ad	6	9–100	150–200 × 5/28 (4)	Stable 40 m after TMZ	PR
	M/45	PRL Ad	2	100	150–200 × 5/28 (5)	Progression	PR
	M/55	PRL Ad	10	20	150–200 × 5/28 (11)	Reduced (66)	Death
	M/60	PRL Ad	2	9	150–200 + CAB (21)	Reduced (80)	Death
	M/68	PRL Ad	NA		150–200 × 5/28	Progression	Death
	M/23	PRL Ad	41	100	150–200 × 5/28 (4)	Progression	Death
	M/22	NF Ad	2	9	150–200 × 5/28 (12)	Reduced (55)	SD 69 m after TMZ
	M/45	NF Ad	2	100	150–200 × 5/28 (18)	Reduced (28)	NA
	F/52	NF Ad	10	90	150–200 × 5/28 (5)	Progression	Death
	M/59	NF Ad	10	90	150–200 × 5/28 (6)	Progression	Death
	M/57	NF Ad	3.3	95	150–200 × 5/28	Progression	Death
	M/51	ACTH Ca	80	0–60	150–200 × 5/28	NA	Death
	M/62	ACTH Ca (NS)	10	95	150–200 × 5/28	NA	Lost to follow-up
	M/70	ACTH Ca	70	9	150–200 × 5/28	NA	NA
	M/46	GH Ca	60	90	150–200 × 5/28	NA	Death
	F/40	GH Ca	20	9	150–200 × 5/28	CR	CR after 48 months
	F/49	PRL-GH Ca	5	9	150–200 × 5/28	CR	CR after 91 months
	F/32	PRL Ca	20	50	150–200 × 5/28	NA	Death
	F/59	PRL Ca	10	NA	150–200 × 5/28	NA	PR

Vieira Neto et al., 2013 [33]	F/54	GH S Ca	2.6	68	150–200 × 5/28	SD	NA

Morokuma et al., 2012 [34]	M/58	NF Ca/NEM-1	7.6	(−)	75/d × 42 days; then 192 × 5/28 + RT (20)	“Visibly declined”	“Improved”

Zhong et al., 2014 [35]	F/30	NF Ad	20	NA	200/d × 5/4 consecutive weeks/2 months + RT (4)	CR	NA

Syro et al., 2009 [36]	M/70	Gn Ad	2–6	30–>50	200 × 5/28 (6)	“Minor reduction” and intratumoral necrosis	Death

Hagen et al., 2009 [37]	F/48	PRL Ad to mixed PRL-GH Ad to Ca	5	(−)	150–200 + CAB/STT-A	Reduced (62)	“Improved”
	M/60	PRL Ad	~2	(−)	150–200 + CAB	Reduced (80)	“Improved”
	M/20	NF Ad	~2	Few (+)	150–200	Reduced (55)	“Improved”

Mendola et al., 2014 [38]	M/58	NS Ca	10	NA	160 × 5/28 (1)	No	No change

Strowd et al., 2015 [39]	F/44	PRL Ad	NA	NA	150–200 × 5/28 (3 months)	“Reduction in tumor size”	PR

Ceccato et al., 2015 [40]	F/67	NF Ad	<3	NA	150–200 × 5/28	Progression	No change
	F/39	GH Ad	<3	NA	150–200 × 5/28	Progression	No change
	M/40	NF Ad	<3	NA	150–200 × 5/28	Decreased (49)	NA
	M/32	ACTH Ad	<3	NA	150–200 × 5/28	Decreased (63)	No change
	M/47	NF Ad → ACTH	<3	NA	150–200 × 5/28 + pasireotide	Decreased (21)	No change

Philippon et al., 2012 [41]	M/41	PRL Ca/MEN-1	NA	NA	200 × 5/28 (24)	Decreased (62)	“Improved”

Fadul et al., 2006 [42]	M/38	NF Ca	1	NA	200 × 5/23 (12)	PR	PR
	M/26	PRL Ca	10		200 × 5/23 (10)	PR	PR

Kovacs et al., 2007 [43]	M/46	PRL Ca	40–60	NA	200 × 5/28 (7)	“Shrinkage”	“Improved”

Cornell et al., 2013 [44]	M/40	ACTH Ad	5–7	NA	200 × 5/28 (3)	Progression	No change

Phillips et al., 2012 [45]	M/25	PRL Ad	23	NA	350 × 5 (1)	No change	Death

Rotondo et al., 2012 [46]	F/49	Crooke cell Ad	5–8	(−)	85 p.o daily + SRT	NA	NA

Arnold et al., 2012 [47]	F/61	ACTH Ca	NA	NA	NA (12)	“Resolved”	PR

Morin et al., 2012 [48]	M/22	GH Ad	3-4	NA	200 × 5/28 (5)	No change	Increased signs

Whitelaw et al., 2012 [49]	M/34	PRL Ad	15	(−)	200 × 5/28 (6)	“Dramatic reduction”	“Significant improvement”
	M/32	PRL Ad	8	(−)	200 × 5/28 (6)	“Substantial reduction”	“Significant improvement”
	M/13	PRL Ad	4	(−)	200 × 5/28 (12)	PR	PR

Ersen et al., 2012 [50]	NA	Gn Ad	NA	Two zones: (−) and (+), 60%	200 × 5/28 (14)	SD	“Clinical improvement”

Scheithauer et al., 2012 [51]	F/13 months	Pituitary blastoma	NA	Varied from 40 to 60%	100 × 5/28 (12 + 6)	Progression	NA

Ortiz et al., 2012 [52]	M/38	ACTH Ad → Ca	NA	High	200 × 5/28 (8)	No change	Progression

Batisse et al., 2013 [53]	M/47	GH Ad	(−)	High	200 × 5/28 (3)	Progression	No significant response

Bruno et al., 2015 [4]	F/52	ACTH Ad	6	(−)	150–200 × 5/28 (29)	CR	CR
	F/42	ACTH Ad	4	(−)	150–200 × 5/28 (12)	CR	CR

Ad: adenoma; Ca: carcinoma; SA: silent adenoma; NS: Nelson's syndrome; NF: nonfunctioning; PRL: lactotrope; ACTH: corticotrope; GH: somatotrope; Gn: gonadotrope; MRI: magnetic resonance imaging; NA: not available; RT: radiotherapy; CR: complete response; PR: partial response; SR: “significant” reduction; METS: metastases; SD: stable disease; CAB: cabergoline; STT-A: somatostatin agonist; CAP: capecitabine; →: change; (og): ongoing.
